# Sleep Deprivation from the Perspective of a Patient Hospitalized in the Intensive Care Unit—Qualitative Study

**DOI:** 10.3390/healthcare8030351

**Published:** 2020-09-21

**Authors:** Katarzyna Lewandowska, Wioletta Mędrzycka-Dąbrowska, Dorota Pilch, Krystyna Wach, Antonietta Fortunato, Sabina Krupa, Dorota Ozga

**Affiliations:** 1 Department of Anaesthesiology Nursing & Intensive Care, Medical University in Gdansk, 80-211 Gdańsk, Poland; kalewandowska@gumed.edu.pl; 2 Zentrum für Anästhesiologie und Intensivmedizin, Universitäts Klinikum Eppendorf, 20251 Hamburg, Germany; pilchdorota@o2.pl; 3 Corso di Laurea in Infermieristica, Università degli Studi di Milano, 20161 Milan, Italy; krystyna.wach@asst-melegnano-martesana.it; 4 Hospital Cernusco sul Naviglio Hospital, 20063 Milan, Italy; antonietta.fortunato@tiscali.it; 5 Department of Emergency Medicine, Faculty of Medicine, University of Rzeszow, 35-359 Rzeszów, Poland; sabinakrupa@o2.pl (S.K.); gdozga@poczta.fm (D.O.)

**Keywords:** sleep deprivation, intensive care unit, sleep, patient’s feelings

## Abstract

(1) Introduction: Sleep architecture of Intensive Care Unit (ICU) patients is altered, with over 60% of them reporting sleep disorders or even sleep deprivation during their stay. The aim of the study was to describe the experiences related to sleep and nighttime rest of patients hospitalized in the ICU. (2) Method: the study used a qualitative project based on phenomenology as a research method. A semi-structured interview was used as the method to achieve the goal. The patients’ answers were recorded and transcribed. The data were coded and cross-processed. (3) Results: twenty-three patients were surveyed, fifteen men and eight women. The average age was 49.7 years. The average time of hospitalization was 34.3 days. During the ICU stay, patients required mechanical ventilation through the tracheostomy tube. Five themes were identified from the interview as factors disturbing sleep: fear, noise, light, medical staff, and at home best. (4) Conclusions: chronic anxiety appears to contribute to sleep disturbances in the ICUs, psychological support, and individualized approach to the hospitalized patient seem necessary. By raising the awareness of the essence of sleep among medical staff, environmental factors can be reduced as disturbing sleep. Based on the participants’ comments, it is possible that repeated actions could also increase the patients’ sense of security.

## 1. Introduction

Sleep is an inseparable and essential element of human life. Adequate amount of undisturbed sleep contributes to the proper functioning of the central nervous system (CNS), the regeneration of the body and the repair of damaged cells [1]. What makes physiological sleep different from similar conditions, such as a coma, hibernation, or anesthesia, is the fast return to wakefulness after a strong stimulus [2]. Physiological sleep and stable sleep/wake cycles are linked to better physical and mental health throughout life. Sleep disturbance is defined as the insufficient amount or quality of sleep with subsequent daytime impairment and significantly reduced general wellbeing [3]. Chronic disturbance of sleep and the natural pattern of sleep and wakefulness relative to the 24-h environmental cycle can lead to various long-term health consequences [4]. A body of research showed a correlation between sleep disturbance and changes in respiratory function parameters [5], increased pain sensitivity, reduced tolerance to fluctuating blood sugar level, as well as increased activity of the sympathetic nervous system [6]. Sleep deprivation related to insufficient sleep before, during and after hospitalization has been rather broadly described in relevant literature [7].

### Background

The Intensive Care Unit (ICU) is the heart of the hospital, where medical staff save lives by treating the most difficult clinical cases. It is a complex environment based on the continuous observation and monitoring of the patient. Given the workload of ICU medical staff, the physical and technical aspects of care become priority, overshadowing the psychological aspects of care and the needs of the patient [8]. During the nighttime, patients of an ICU are exposed to continuous noise made by the monitoring devices, light and frequent medical interventions. Due to the specificity of the ward, medical personnel are not able to eliminate the factors responsible for poor sleep quality and lack of physiological rest [9].

The sleep architecture of ICU patients is altered, and in some studies over 60% of ICU patients reported sleep disorders or even sleep deprivation during their stay [10]. The available diagnostic measures confirmed that 90% of patients treated for critical conditions experienced poor sleep quality with numerous sleep disruptions throughout the cycle [11]. First studies focusing on sleep disturbance in ICU patients were conducted in the 1970s and measured sleep quality by means of polysomnography. The studies found that patients experienced sleep disturbance, decrease in total sleep time (TST), and changes in sleep architecture. Stages N1 and N2 of Non-Rapid Eye Movement (NREM) sleep were extended, while N3 stage of NREM and Rapid Eye Movement (REM) sleep were reduced. The cited studies were limited to 8 h of night sleep, with mechanically ventilated and sedated patients excluded from the project [12]. Other studies showed that patients recalled frequent sleep interruptions as well as feelings of pain, anxiety, and fear, all of which impaired their ability to sleep during their stay in an ICU [13,14,15]. A study carried out on 464 patients found that 51% of patients experienced dreams and nightmares, with 14% of them declaring that nightmares continued to impact the quality of their lives 6 months after their stay in an ICU. Another study conducted in 2008 showed that within a 24-h stay in the ICU, patients experienced 41 ± 28 episodes of sleep, which lasted a maximum of 15 ± 9 min. Thus, their sleep was marked by high fragmentation and low efficiency [10].

## 2. Methods

This study was aimed at describing the experiences of patients hospitalized in intensive care units related to sleep and nighttime rest and the associated impact on their sleep following their discharge from the hospital. The study used qualitative approach and relied on phenomenology, which is a research method used to study experience as experienced from the subjective point of view. As such, phenomenology is the foundation of accurate nursing research. It appreciates individual perspective and the relationship between nurses and patients, offering holistic approach to a person [16]. Phenomenology was used in this study as a research method to describe experiences of patients of an ICU related to nighttime rest. Understanding patient perspective is key to showing that their experience is real, important and lived.

### 2.1. Sample and Recruitment

**Figure 1:**
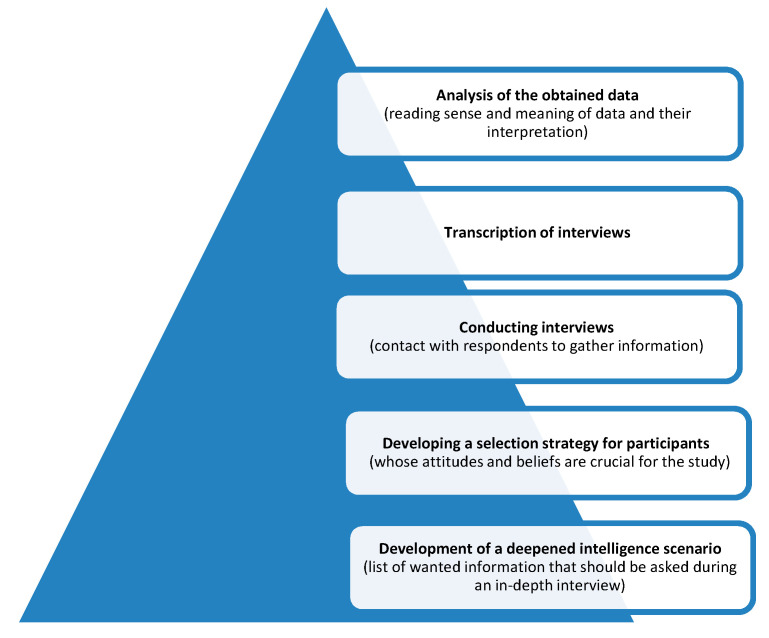
Stages of conducting the study using in-depth interview.

The study was carried out in an academic hospital and lasted throughout 2019. This study was approved by the ethics committee. Each patient received an explanation about the purpose of the study and gave oral consent prior to their participation. Anonymity and confidentiality were also assured. Patients were recruited by nurses/doctors from the General ICU and the Cardiac Surgery ICU. The study included patients who stayed in an ICU for at least 7 days, including the last 7 days without pharmacological sedation. They had to be at least 18 years old, speak Polish, and express their conscious consent to be included. Initially, the patients were approached by a third party—an ICU nurse—who informed them of the study and provided them with the researcher’s contact details. Out of fifty patients asked to participate, twenty-three agreed. The research was carried out in accordance with the principles of conducting in-depth interviews (Figure 1).

### 2.2. Questionnaire Development

**Figure 2:**
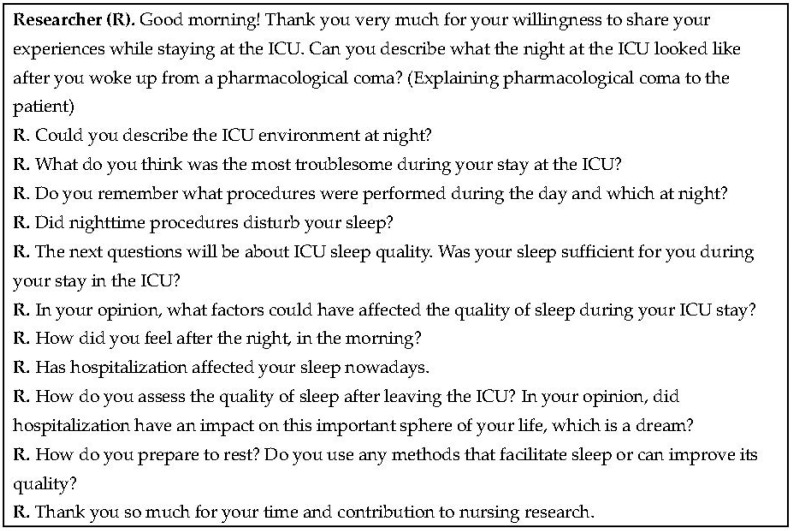
Interview using a questionnaire comprising of ten questions.

A semi-structured interview was used as the method to meet the research objectives [17]. The conversation with the patient, carried out by two researchers, was based on a questionnaire composed of 10 questions (Figure 2). The form of the interview made it possible for the interviewers to ask follow-up questions, when additional information was required. The researchers had previous experience in conducting in-depth interviews and were able to stay patient and open-minded throughout each interview.

### 2.3. Data Collection

The researchers discussed the topic and aim of the study with the subjects. Each of them expressed their consent for the interview to be recorded. Interviews were conducted by two researches, after the patient’s discharge from the ward. The location of the interview depended on a participant’s wishes: two subjects were interviewed in a public place (a café), and three in their homes, with the remaining interviews taking place in the hospital café. At the beginning of each interview, the researchers introduced the former ICU patients to the topic and got to know them in order to create a sense of trust. As a next step, the researchers asked the prepared questions and recorded the answers. The interviews ended with a summary and a conversation about a subject’s recollections of his/her stay in an ICU. The interviews lasted for an hour on average. The researchers tried to be aware of the openness that is crucial for the phenomenological approach. The open approach means that the researcher opens up to the phenomenon as it presents itself [17]. Such an attitude is reflected in the researcher’s receptive, perceptive, sensitive, and reflective attitude [18]. Being open-minded was about putting everything else aside and dedicating yourself to listening and understanding the interlocutor’s relationship with their patients experience. When the participants avoided talking about certain issues, started feeling awkward, ashamed, or became very emotional, the interviewer stopped the interview for ethical reasons [17].

### 2.4. Data Analysis

First, the patient was assigned a code based on the date and time of the interview. Then the head researcher transcribed and analyzed the research material using phenomenology. Each respondent was assigned a number in accordance with the chronology of interviews. The assigned numbers were used during qualitative analysis. For the purposes of this article, the letter ‘P’ (patient) was added to each number. The respondent has been personalized. Labels with the letter ‘P’ were used to protect the subjects’ identities.

Thematic analysis was carried out based on the holistic, selective and detail-oriented approach offered by van Manen [19]. As proposed by the scholar, the researcher listened to the recordings while simultaneously reading the transcription to identify any inconsistencies. The literal transcriptions or respondents’ written answers were read and reread. Each record was read in full at least three times. A respondent’s statements explaining his/her experience of ICU were highlighted (selectively). These included thematic statements reflecting his/her experience. In order to better understand the respondent’s perception of his/her experience of an ICU, the researchers applied the ‘close-read’ approach, which means that each sentence or sentence group in each of the interviews was thoroughly analyzed (line-by-line analysis with focus on detail). The goal of this analysis method was to uncover the essence of the experience. The respondent’s narrative accounts and interpretative summaries were coded to help identify the themes emerging from the collected data. It was found that the respondents’ accounts were interconnected and consistent. The same themes were identified in all respondent groups. Data triangulation yielded five integrating common themes: “anxiety,” “noise,” “light,” “medical staff” and “there’s no place like home.” Some of the themes were dropped if they did not fit well with the emerging structure or because they had a weak evidential base [17].

## 3. Results

Twenty-three patients, fifteen men, and eight women took part in the study (Table 1). The average age was 49.7 years. The average time of hospitalization was 34.3 days. During the ICU stays, patients required mechanical ventilation through the tracheostomy tube. ICU had an open architectural structure.

### 3.1. Anxiety

While describing their experiences, each of the subjects reported anxiety-related components. They were diverse, depending on individual concerns. For example, patient (P1) expressed his anger with himself and fear that he would not return to his full former condition.

*“Stress wasn’t as much of a nuisance as the anger and fear that I might not return to my full former condition. This also had a huge impact on my sleep, because deep down I kept thinking that things would never be the same again, and such thoughts came to me when I was asleep.”*

(P1)

Patient (P2) displayed the fear of a new situation and the impossibility to adapt to hospitalization. She also experienced the fear of death and concern about the future.

*“There was one time when I was woken up from my coma, and I had that feeling of suffocation and I found it hard to sleep. I was afraid to fall asleep for fear that I wouldn’t wake up again. I panicked… I had to be given oxygen although I didn’t really need it.”*

(P2)

Another patient’s anxiety was caused by her being unable to call for help—she feared for her life and was overwhelmed by a sense of loneliness. Her inability to adapt to the situation as it was and a fear of the unknown were also visible.

*“I don’t know if the fear of surgery was worse or anxiety or if everything is good when I woke up after the surgery. I had this endotracheal tube and I couldn’t ask anyone. After a while, the nurse said that she was already after the surgery, but I did not know this place and these people. Maybe if I had seen this room before, it would have been better for me.”*

(P9)

An additional factor disturbing sleep was the reversal of circadian rhythm. As a consequence, patients experienced anxiety related to a lack of sense of time.

*“After waking up, I couldn’t bear to lie down. I felt thirsty, I wanted to roll on the bed, but no one understood what I was saying. Night? I thought it lasted forever. All in all, it was only during the visit that I saw the watch and I knew it was morning. There all the time I was unsure what time it is.”*

(P8)

### 3.2. Noise

The ICU environment in its various forms was mentioned as a factor disturbing the patients’ sleep and nighttime rest. Noise of various types was reported most frequently. The first patient described noise as a factor making it impossible for her to fall asleep and waking her up.

*“Noise did nothing but disturb; those loud conversations between nurses, stamping, pushing things. (…) I don’t think that made it easier for me to fall asleep, but made it all the more difficult. (….) And there were those situations when at five o’clock in the morning (although they wake patients up probably at 8 for the morning hygiene routines) the nurse would give me my medications, but two or three of them would get together by the isolation room and they would talk by my bed, wake me up and so on; this was what would wake me up—their conversations.”*

(P1)

In the case of another patient, the impossibility to sleep was related to alarms from a cardio-monitor, which pierced the night silence and woke him up.

*“The worst thing about staying at the ICU was that you never know what noise will wake you up. Sometimes it was so that for 3 h it was quite quiet, and immediately began to turn on the pumps, you could hear metal baskets. Sometimes you sleep there an hour, sometimes 10 min and sometimes not at all. You never know what the night will bring.”*

(P8)

### 3.3. Light

Another environmental factor disturbing the patients’ nighttime rest was light: both the artificial night light enabling the nursing staff to work, and the daylight preventing the patients from catching up on sleep during the day.

*“The exhausting light: natural light during the day, and artificial light at night. At some point I asked my family for sunglasses (…) so when I could sleep during the day because there was only slight noise in the background but I was disturbed by the light, I would lie wearing them and fall asleep. The nurses laughed at me, but they always helped me to put them on, because my paralysis stopped me from doing so.”*

(P4)

Patient (P1) describes light as a factor, which woke him up: a light suddenly being turned on disrupted his sleep and made it impossible for him to fall asleep again.

*“During the evening, nurses tried especially to dim the light in the room but all of a sudden, at 8:00 p.m., there was a round and they turned all the lights on in the room as they had to read all the records. I felt dizzy as though I’d been woken by a brutal force. And it was always like that whenever they intervened during the night; when something was going on with other patients, they always turned the light on in the entire room. It was awful.”*

(P1)

### 3.4. Medical Staff

The medical staff and the care and treatment activities undertaken were identified as yet another barrier to proper rest and sleep for patients in an ICU. The pace of work and the frequent daily routine in patient care had a negative effect on night rest

*“I felt like I was the bed number. Everyone was saying to each other, Have you looked at seven? There was a pump to change. But when the nurses came to bed I felt like I was finally getting my name back. Then I felt safe and important at the same time. I could fall asleep safely.”*

(P18)

Sleep disturbance was also caused by interventions to do with other patients. Due to the ICU environment, actions undertaken in relation to given patients disturbed the other hospitalized patients.

*“Well… what was most annoying for me was when nurses or doctors intervened with other patients who were around me; I always automatically woke up when something was going on in my room.”*

(P19)

*“There was a patient next to me who had to have cardioversion several times during the night, and all that commotion was stressful for me, so I obviously couldn’t sleep either.”*

(P3)

### 3.5. There’s No Place like Home

The factor affecting sleep disorders was longing for the family, the lack of communication with them.

*“Then I remember that the nurse answered my daughter’s phone and said I was still asleep and I had a respirator. And I wanted to say everything was OK, but I couldn’t. Then I was worried all the time if my family knew I was doing well.”*

(P10)

All of the subjects evaluated their sleep after hospitalization as good. The quality of their sleep improved when they left the hospital environment and returned to their own rhythm. It is worth pointing out that all of the patients reported extreme exhaustion connected with prolonged absence of the appropriate amount and quality of sleep.

*“(…) I didn’t feel regenerated at all, I felt more exhausted each day. I had no appetite, my meals were irregular, I felt like I was on medication all the time and I didn’t feel regenerated even after a night’s sleep. Regardless of whether I slept during the night or not, I didn’t feel regenerated.”*

(P1)

*“I didn’t have nightmares to do with the ICU or the accident. When I returned home, I slept very well to regenerate.”*

(P3)

Sleep disturbance experienced after the patients’ discharge from hospital is also related to the reason behind their hospitalization. As a result of multiple trauma, patient (P4) lost his right lower limb. He recalled the pain, which made it impossible for him to sleep at night.

*“I already sleep well—I sleep poorly when I feel pain in my leg stub—after an active day.”*

(P4)

It should also be mentioned that during the interviews the subjects were also asked what could improve the quality of sleep at an ICU. The needs were personalized. In the light of the shortfalls, each of the patients expressed their suggestion for an improvement in the sleep quality in ICU conditions. Patient (P5) exhibited a holistic approach and summarized the topic providing suggestions of key importance:

*“(…) It seems to me that it’s a question of every patient requiring different things individually. I think that when one patient needs peace, another will sleep well.”*

(P5)

## 4. Discussion

Sleep architecture is altered in ICU patients. All study subjects experienced sleep disturbance and were not satisfied with their nighttime rest [1,8,11]. Their ability to sleep was most affected by fear-related factors. A strong sense of powerlessness and helplessness related to their situation led to sleeping difficulties. A similar study carried out in 2013 showed that ICU patients perceived quality sleep as a condition for recovery and a sign of returning to health [20]. Some of them were afraid to sleep due to recurring nightmares, which led to constant tiredness and anxiety. The death of a fellow patient staying in the same ward also contributed to feelings of insecurity, causing study subjects to experience sleep difficulties. They were too frightened to sleep in a place in which death was a common occurrence. Research from 2013, which also analyzed the feelings of ICU patients, confirmed the link between a fellow patient’s death and sleep disturbance: “*I couldn’t sleep because people were dying around me.*” The patient remained awake, since it made him feel safe [20].

Noise was identified as another significant factor affecting sleep. Patients were disturbed by cardio monitors, conversations between medical staff, and interventions concerning other patients. Analyses show that among factors affecting sleep quality in ICU patients, noise is the dominant one [21]. In accordance with the guidelines issued by the World Health Organization, noise level should not exceed 30 dBA. ICU patients are exposed to noise levels at 53–59 dBA on average, on a daily and nightly basis, with peak noise levels reaching as high as 67–86 dBA [22]. Moreover, non-ventilated patients reported noise (53.3%) as the main barrier to sleeping in an ICU [23].

Another important factor disturbing nighttime sleep was light. In an ICU, the exposure of patients to sunlight is limited and artificial light is insufficient to effectively stimulate the circadian rhythm. The inappropriate intensity of light may affect circadian stimulation and have an adverse effect on the duration of sleep [5,8]. Light measurements carried out in four intensive care units showed that the average maximum level of nighttime light intensity ranged from 128 to 1445 lux, which is high enough to suppress melatonin production, but not high enough to disturb sleep. Regardless of light levels, nocturnal melatonin secretion in ICU patients is disturbed or suppressed, suggesting that factors other than light and darkness affect the circadian rhythm in this population [5].

Medical personnel interventions concerning the study subjects as well as patients in the same room were identified as another factor directly disturbing sleep. Patient care activities in an ICU can cause between 40 and 60 interruptions during sleep [5]. Bilhari et al. assessed the extent to which nursing interventions interfere with patients’ sleep, using a 10-point scale (1 = worst sleep quality; 10 = best sleep quality). The respondents assessed the effect of nursing interventions on sleep of ICU patients at 3.5/10 [21]. Incidentally, patients considered the presence of nursing staff to be a factor increasing their sense of security [8]. Research carried out in 2013 determined that as many as 70.6% of the subjects linked sleep disturbance to the fact that they did not know the names of the nursing staff with whom they interacted. The inability to understand medical terminology was responsible for additional concern [24].

Research showed that after the patients were discharged from the hospital, sleep disturbance developed during their ICU stay continued for a limited length of time. Patients reported that they soon returned to systematic sleep pattern and hygiene they followed before hospitalization. McKinley et al. presented contrary conclusions, indicating that a group of patients hospitalized in an ICU displayed mental disorders, symptoms of anxiety and high stress up to 8 weeks following their discharge. The research also found that over time patients’ sleep improved [25].

It is important to increase the awareness of the essence of the problem among ICU staff. In the new 2018 guidelines on pain, agitation/sedation, delirium, immobility, and sleep distribution, the Critical Care Society recommends using a protocol that improves sleep quality [26]. By applying the same recommendations for the use of eye protection bands to avoid light or earplugs to reduce noise, medical staff can provide better care to patients [26]. Staff education is key to increasing the quality of sleep in ICU patients. Evidence suggests that raising the awareness of the therapeutic team and improving the cooperation among its members can minimize sleep disturbance.

Sleep quality of an ICU patient can be improved by increasing patient’s comfort, reducing nighttime light and noise levels, stepping up the management of patient care process, and clustering nursing and care-related activities to provide uninterrupted periods of sleep. Noise can be reduced by adjusting the alarms of monitors and respirators, minimizing staff conversations, and closing the doors to patients’ rooms.

## 5. Conclusions

This study suggests that anxiety is a contributing factor in sleep disturbance. Medical teams may find that recruiting psychologists who are instrumental in helping patients deal with the situation can minimize the stress that comes with staying in an ICU. Other factors related to the ICU environment contributing to sleep disorders include noise, light, and medical personnel. Every patient has his/her own individual sleep hygiene. Identifying a patient’s sleeping pattern and adjusting the environment accordingly may play a key role in ensuring nighttime comfort. The development of guidelines facilitating the identification of sleep disturbance of ICU patients could standardize the help provided to them and improve the quality of nighttime rest.

## 6. Implications for Nursing and Health Policy

Raising medical staff awareness of the importance of nighttime rest is key to improving the quality of sleep in ICU patients. With the daily care provided by therapeutic teams being so demanding, patients’ rest and sleep quality become of secondary importance. However, if medical staff’s attention is turned to the benefits of physiological rest and the importance of the regeneration of the body, they could adopt a comprehensive approach to patient’s nighttime sleep and see it as yet another element of patient’s wellbeing, which needs to be monitored on a regular basis. The development of a standard and guidelines addressing sleep disturbance may help to standardize the implemented measures.

As a basic measure to minimize sleep disturbance, nighttime rest should be recognized as a fixed element of patient care and, in the case of a stable patient, a time window should be selected when the number of nursing interventions could be reduced to a minimum. The regularity of repetitive procedures would have the additional benefit of increasing patients’ sense of security.

In addition, steps should be taken to reduce anxiety, which leads to severe sleep disturbance. The help of a psychologist may be an effective interdisciplinary measure of reducing stress experienced by patients. Their mental health may also benefit if they are provided with emotional support and basic information to help them understand the procedures they are undergoing.

## 7. Limitations

There is a big problem with access to patients hospitalized in ICUs. Due to the tightening of legal aspects related to the protection of personal data, it is difficult to establish cooperation with the patient after the period of hospitalization. The time of stay at the ICU is very exhausting and traumatizing for patients who do not want to come back with memories until then.

## Figures and Tables

**Table 1:** Basic data of patients undergoing testing. ICU, Intensive Care Unit.

No. Patient	Age	Sex	The Reason for Hospitalization	Time of Stay in ICU (Days)	Has the Patient Had Tracheostomy	Was the Patient in the General/Isolated Room
P1	23	Male	multifocal injury	14	no	general room
P2	36	Female	multifocal injury, transport accident	88	yes	isolated room
P3	28	Female	multifocal injury, transport accident	28	yes	general room
P4	33	Male	multifocal injury	56	yes	isolated room
P5	34	Male	cervical spine injury	32	yes	general room
P6	42	Male	cardiac surgery—by pass	12	no	general room
P7	55	Female	cardiac surgery—aortic dissection	45	yes	isolated room
P8	61	Male	cardiac surgery—aortic valve plastic surgery	16	no	general room
P9	47	Female	cardiac surgery—aortic valve plastic surgery	14	no	general room
P10	49	Female	cardiac surgery—aortic dissection	18	no	general room
P11	27	Male	cardiac surgery—aortic valve plastic surgery	7	no	general room
P12	39	Male	cardiac surgery—mitral valve replacement	9	no	general room
P13	53	Male	cardiac surgery—aortic dissection	7	Yes	general room
P14	46	Female	cardiac surgery—by pass	42	yes	isolated room
P15	50	Male	cardiac surgery—aortic dissection	75	yes	isolated room
P16	74	Male	respiratory failure	112	no	general room
P17	90	male	respiratory failure	7	no	general room
P18	71	female	respiratory failure	126	yes	isolated room
P19	78	Male	cardiac surgery—aortic dissection	21	no	general room
P20	89	female	respiratory failure	7	no	general room
P21	29	Male	multifocal injury	21	no	isolated room
P22	32	male	cervical spine injury	14	no	general room
P23	58	male	multifocal injury	18	no	general room
